# Error-Related Activity in Striatal Local Field Potentials and Medial Frontal Cortex: Evidence From Patients With Severe Opioid Abuse Disorder

**DOI:** 10.3389/fnhum.2020.627564

**Published:** 2021-02-01

**Authors:** Elena Sildatke, Thomas Schüller, Theo O. J. Gründler, Markus Ullsperger, Veerle Visser-Vandewalle, Daniel Huys, Jens Kuhn

**Affiliations:** ^1^Department of Psychiatry and Psychotherapy, Faculty of Medicine and University Hospital Cologne, University of Cologne, Cologne, Germany; ^2^Center for Military Mental Health, Military Hospital Berlin, Berlin, Germany; ^3^Department of Psychology, Otto-von-Guericke University, Magdeburg, Germany; ^4^Center for Behavioral Brain Sciences, Magdeburg, Germany; ^5^Department of Stereotactic and Functional Surgery, Faculty of Medicine and University Hospital Cologne, University of Cologne, Cologne, Germany; ^6^Department of Psychiatry, Psychotherapy, and Psychosomatic Medicine, Johanniter Hospital Oberhausen, Oberhausen, Germany

**Keywords:** performance monitoring, local field potential, error processing, error-related negativity, error positivity, nucleus accumbens, intracranial recordings, deep brain stimulation

## Abstract

For successful goal-directed behavior, a performance monitoring system is essential. It detects behavioral errors and initiates behavioral adaptations to improve performance. Two electrophysiological potentials are known to follow errors in reaction time tasks: the error-related negativity (ERN), which is linked to error processing, and the error positivity (Pe), which is associated with subjective error awareness. Furthermore, the correct-related negativity (CRN) is linked to uncertainty about the response outcome. Here we attempted to identify the involvement of the nucleus accumbens (NAc) in the aforementioned performance monitoring processes. To this end, we simultaneously recorded cortical activity (EEG) and local field potentials (LFP) during a flanker task performed by four patients with severe opioid abuse disorder who underwent electrode implantation in the NAc for deep brain stimulation. We observed significant accuracy-related modulations in the LFPs at the time of the ERN/CRN in two patients and at the time of Pe in three patients. These modulations correlated with the ERN in 2/8, with CRN in 5/8 and with Pe in 6/8, recorded channels, respectively. Our results demonstrate the functional interrelation of striatal and cortical processes in performance monitoring specifically related to error processing and subjective error awareness.

## Introduction

Striving for fast action performance is prone to behavioral errors and requires increased cognitive control to avoid them. Successful action adaptation is dependent on the performance monitoring system, a cognitive function that constantly monitors actions and their respective outcomes. If necessary, this system initiates behavioral adaptations to improve performance and to prevent errors.

The performance monitoring system comprises the cortical and subcortical regions (Ullsperger et al., 2014a). Specifically, the posterior medial frontal cortex (MFC) and its sub-region, the anterior midcingulate cortex (aMCC), have been linked to various functions in performance monitoring, such as goal-oriented behavior and error and conflict detection (Ullsperger and von Cramon, 2001; Holroyd et al., 2004; Ullsperger et al., 2014a). Utilizing electroencephalography (EEG), several event-related potentials that correlate with distinct processes of performance monitoring have been identified. The error-related negativity (ERN) is a mid-frontal negative deflection peaking at 50–150 ms after an incorrect response (Falkenstein et al., 1990, 2000; Gehring et al., 1993). The ERN is generated in the posterior MFC and thought to index the need for cognitive control (Ullsperger et al., 2014a,b). An ERN-like but smaller deflection is frequently observed in correct trials (Falkenstein et al., 2000; Coles et al., 2001). This correct-related negativity (CRN) seems to arise by uncertainty about the response outcome and likely initiates behavioral adjustments to improve performance (Bartholow et al., 2005; Ullsperger et al., 2014a). The ERN is followed by the error positivity (Pe), a positive deflection 200–500 ms after the response that is associated with subjective error awareness (Falkenstein et al., 1990, 2000; Wessel et al., 2011; Murphy et al., 2012; Grutzmann et al., 2014) and is putatively generated by sources in the parietal cortex and rostral aMCC (Van Veen and Carter, 2002; Herrmann et al., 2004).

The subcortical components of the performance monitoring system extend over a large network, including basal ganglia structures like the nucleus accumbens (NAc) (Ullsperger and von Cramon, 2006). Given the importance of the NAc in performance monitoring, reward system, and its role as a limbic–motor interface, it has become an important target structure for the treatment of psychiatric disorders with deep brain stimulation (DBS), especially obsessive–compulsive disorder (Huys et al., 2019; Denys et al., 2020) and substance use disorders (Kuhn et al., 2011, 2014; Valencia-Alfonso et al., 2012). The NAc is anatomically and functionally connected to the MFC (Haber et al., 2000; Haber and Knutson, 2010). Particularly, the ERN was abolished by striatal lesions (Ullsperger and von Cramon, 2006) and enhanced by NAc high-frequency stimulation (Kuhn et al., 2011). Furthermore, an ERN-like local field potential (LFP) was found in the NAc following error commission (Munte et al., 2007; Heinze et al., 2009).

We aimed to further characterize the interrelation of NAc and ERN/CRN and Pe in a sample of patients with opioid use disorder who are part of a clinical trial evaluating the clinical efficiency of NAc DBS. The implantation of depth electrodes for DBS treatment provides a unique opportunity to simultaneously record LFPs and cortical EEG. We employed the well-established flanker task that requires the engagement of the performance monitoring system and reliably elicits the ERN and Pe (e.g., Falkenstein et al., 2000; Danielmeier et al., 2009). We expected to find an ERN-like and a Pe-like modulation of NAc-LFPs by response errors (Munte et al., 2007; Kuhn et al., 2011). To assess the relationship between EEG and NAc-LFPs, we performed correlation analyses.

## Methods

### Patients

Four patients (P, see Table 1) with long-lasting opioid abuse disorder, participating in the clinical trial “Deep Brain Stimulation of the Nucleus Accumbens as a Novel Treatment in Severe Opioid Addiction (NASA)” (ClinicalTrials.gov ID: NCT01245075), received bilateral implantation of depth electrodes in the NAc for subsequent DBS. The results of the clinical study have not been published yet (total *n* = 4). All patients were using levomethadone as part of their substitution therapy.

**Table 1:** Demographic data of the patients.

	**Gender**	**Age**	**Handedness**	**Levomethadone (mg)**	**BDI**
P1	M	51	Right	30	31
P2	M	57	Right	30	5
P3	M	46	Right	50	33
P4	M	24	Right	15	39

This study was carried out in accordance with the recommendations of the ethics committee of the University of Cologne and with written informed consent from all the subjects. All the patients gave written informed consent in accordance with the Declaration of Helsinki. The protocol was approved by the ethics committee of the University of Cologne.

### Localization

**Figure 1:**
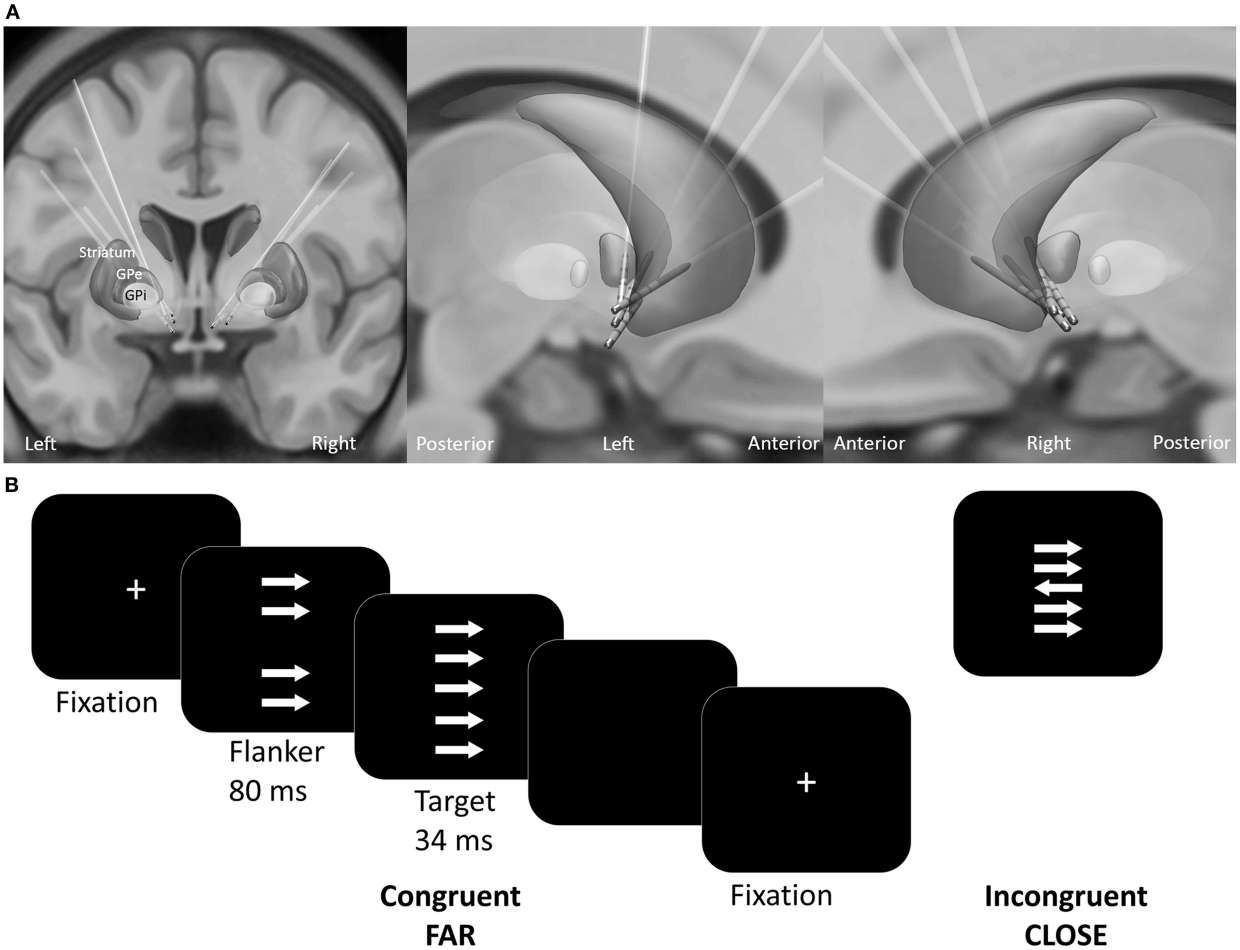
**(A)** Reconstructed electrode location of all patients in MNI space. **(B)** Experimental design of the custom version of the Erikson Flanker task. Patients had to respond accordingly to the target arrow orientation with a button press. The conditions varied regarding the congruency (congruent, incongruent) and distance (far, close) between the target arrow and the flankers.

Electrodes were localized using Lead-DBS toolbox (1.6.5.1) (Horn and Kühn, 2015). Briefly, preoperative T1-weighted MRI images were co-registered with postoperative CT images using SPM 12. The images were normalized into MNI-ICBM 2009b NLIN asymmetric space using the SyN registration approach as implemented in the Advanced Normalization Tools (Avants et al., 2008). The DBS electrodes were automatically pre-localized in MNI space and corrected manually if needed. All electrodes are visualized in Figure 1A.

### Experimental Design

The patients performed a customized arrow version of the Eriksen flanker task [(Eriksen and Eriksen, 1974; Danielmeier et al., 2009); Presentation v10.3, Neurobehavioral Systems Inc., Albany, U.S.A.]. Patients responded to the orientation of the target arrow by pressing a button as quickly and accurately as possible. On each trial four distractor stimuli (flankers) appeared 80 ms before target presentation (Figure 1B). The flankers had either the same (congruent) or the opposite orientation (incongruent stimuli) relative to the target. The distance between the target and the flankers varied between close (3.5° and 1.75° of visual angle below and above the center) and far (6.5° and 4° of visual angle below and above the center). To ensure comparable accuracy-speed tradeoff a colored frame instructed the patient to speed up, to slow down or to maintain current speed level. The response-stimulus interval was between 750 and 1,250 ms. A total of 800 trials were equally distributed across four conditions: (1) far compatible, (2) far incompatible, (3) close compatible, (4) close incompatible. The trials were presented in a pseudo-randomized order in blocks of 100 trials. Patients received no performance feedback.

### Data Collection

The sessions took place in a dimly lit and electrically and acoustically shielded chamber 1–3 days after electrode implantation. LFPs were recorded from externalized quadripolar depth electrodes (Medtronic 3389; Medtronic, Inc., Minneapolis, MN, USA), with a lead contact width of 1.5 mm, diameter of 1.27 mm, and distance between contacts of 0.5 mm. Au electrodes (between 11 and 20) were placed individually according to the extended 10–20 system (Jasper, 1958) for scalp recordings. The electrode placement varied due to surgical incisions, but electrodes FCz and CPz were always included. All data were recorded at a sampling rate of 5,000 Hz (BrainAmp MR plus amplifiers; Brain Products, Gilching, Germany), and all impendances were kept below 15 kΩ.

### Data Analysis

The data were first processed using Brain Vision Analyzer (Brain Products GmbH, Germany). The data were band pass-filtered (48 db/Oct, Butterworth; zero-phase IIR filter) between 0.5 and 40 Hz and resampled to 500 Hz. The data were further processed using EEGLAB 10.2.5.8b (Delorme and Makeig, 2004) and custom Matlab 7.13 routines (The Math Works Inc., Natik, USA). LFPs were obtained by re-referencing all available contacts against each other. This resulted in maximally six bipolar channels per electrode. Epochs were rejected when exceeding five standard deviations of the joint data probability. Artifacts (eye movements, pulse artifacts) were identified by using independent component analysis (4 ± 1 rejected independent components) (Makeig et al., 2004) and subsequently rejected. Scalp data were re-referenced to linked mastoids. Response-locked epochs were extracted from −1,300 to 1,500 ms. Baseline correction was applied from −300 to −100 ms relative to stimulus onset. Trials with reaction times (RT) shorter than 150 ms or exceeding 1,000 ms and trials containing response correction within 150 ms after the initial response were excluded from the analysis. EEG data were analyzed only for incompatible trials due to the expected insufficient number of compatible error trials. To increase signal-to noise ratio in EEG analysis, we collapsed across close and far conditions (Fischer et al., 2017). This resulted in at least 44 incompatible error trials (mean, 79 ± 22) and 217 incompatible correct trials (mean, 296 ± 26) per patient. The ERN and CRN amplitudes were defined as mean amplitudes at electrode FCz ±40 ms centered around the grand average peak (search window 0 to 150 ms) (Fischer et al., 2017). The Pe was defined as the mean amplitude from 200 to 500 ms following an erroneous response at electrode CPz (Falkenstein et al., 2000).

For the LFP analysis, we included one channel per hemisphere that displayed the greatest variance over the whole epoch. The mean LFP amplitudes were calculated using the same time windows as for the ERN/CRN and Pe, respectively. For improved readability, these LFPs are referred to as ERN-LFP, CRN-LFP, and Pe-LFP hereafter.

### Statistical Analyses

Analyses were performed at the single-trial level (within-subject) due to the small sample size. The putative effects of COMPATIBILITY on error rates were examined with a Pearson Chi-square test. Single-trial RTs were analyzed using ANOVA with the factors ACCURACY (error, correct) and COMPATIBILITY (compatible, incompatible). Single-trial EEG and LFP amplitudes were compared between error and correct trials using two-tailed *t*-tests.

Spearman correlations of ERN, CRN, and Pe amplitude with respective LFP amplitudes in the NAc were performed using single-trial data.

Correction for multiple comparisons was applied using false discovery rate. Corrected *p*-values are reported.

## Results

### Behavior

**Figure 2:**
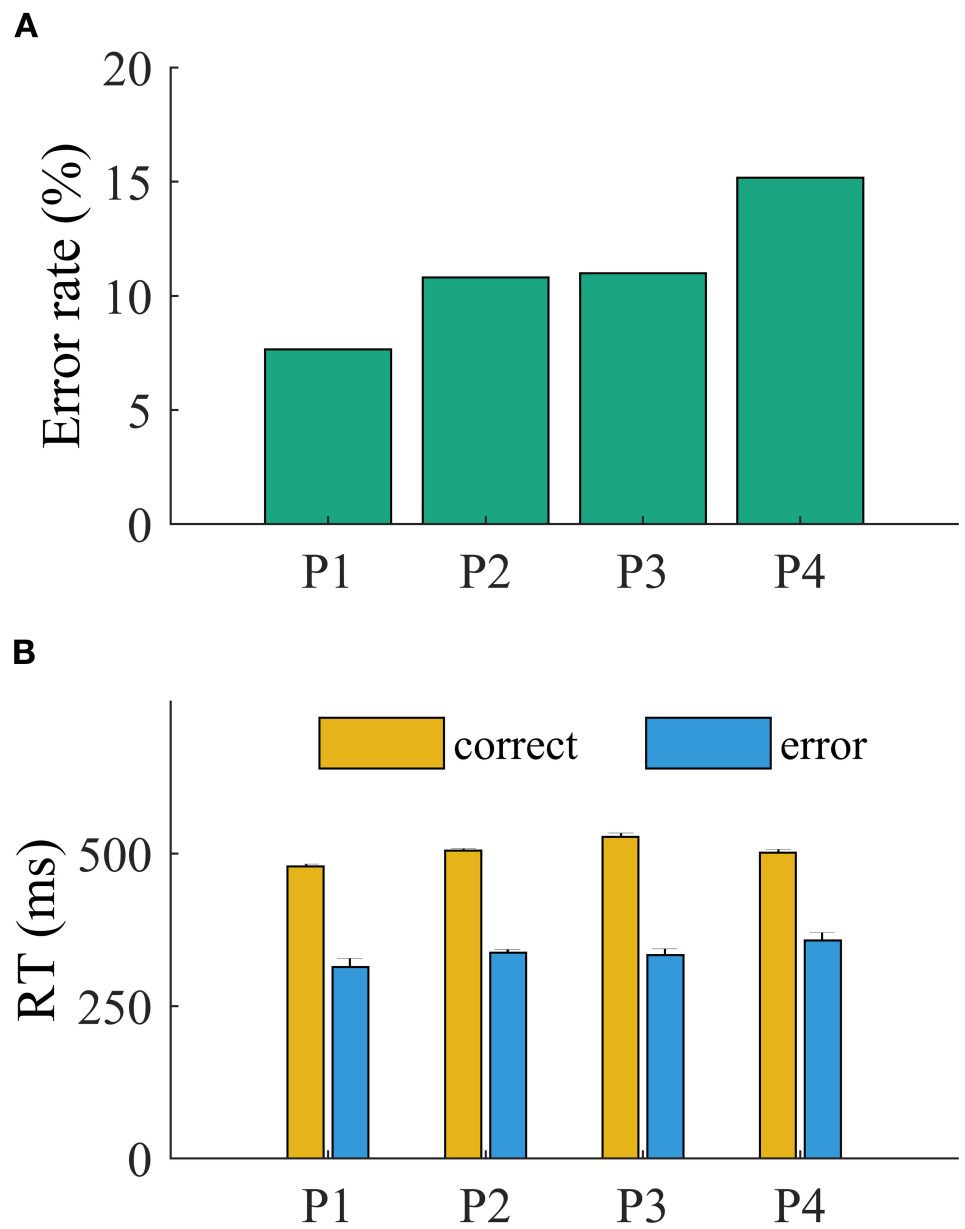
Behavioral results. **(A)** Error rate and **(B)** overall reaction time.

The patients committed more errors in incompatible than in compatible trials (all *p* < 0.001, Pearson chi-square test). The ANOVA revealed a main effect of accuracy on RT (all *p* < 0.001). The RTs were faster for error (mean, 350 ± 11 ms) than for correct trials (mean, 456 ± 8 ms; all *p* < 0.001, *t* < −11.95; Figure 2). The main effect of compatibility was significant for P3 [*F*_(1,781)_ = 6.7, *p* = 0.039; all other *p* > 0.1]. The interaction of accuracy and compatibility was significant for all patients (*p* < 0.05), indicating that the slowing of RT is driven by incompatible trials.

### Electrophysiological Results

#### EEG

**Figure 3:**
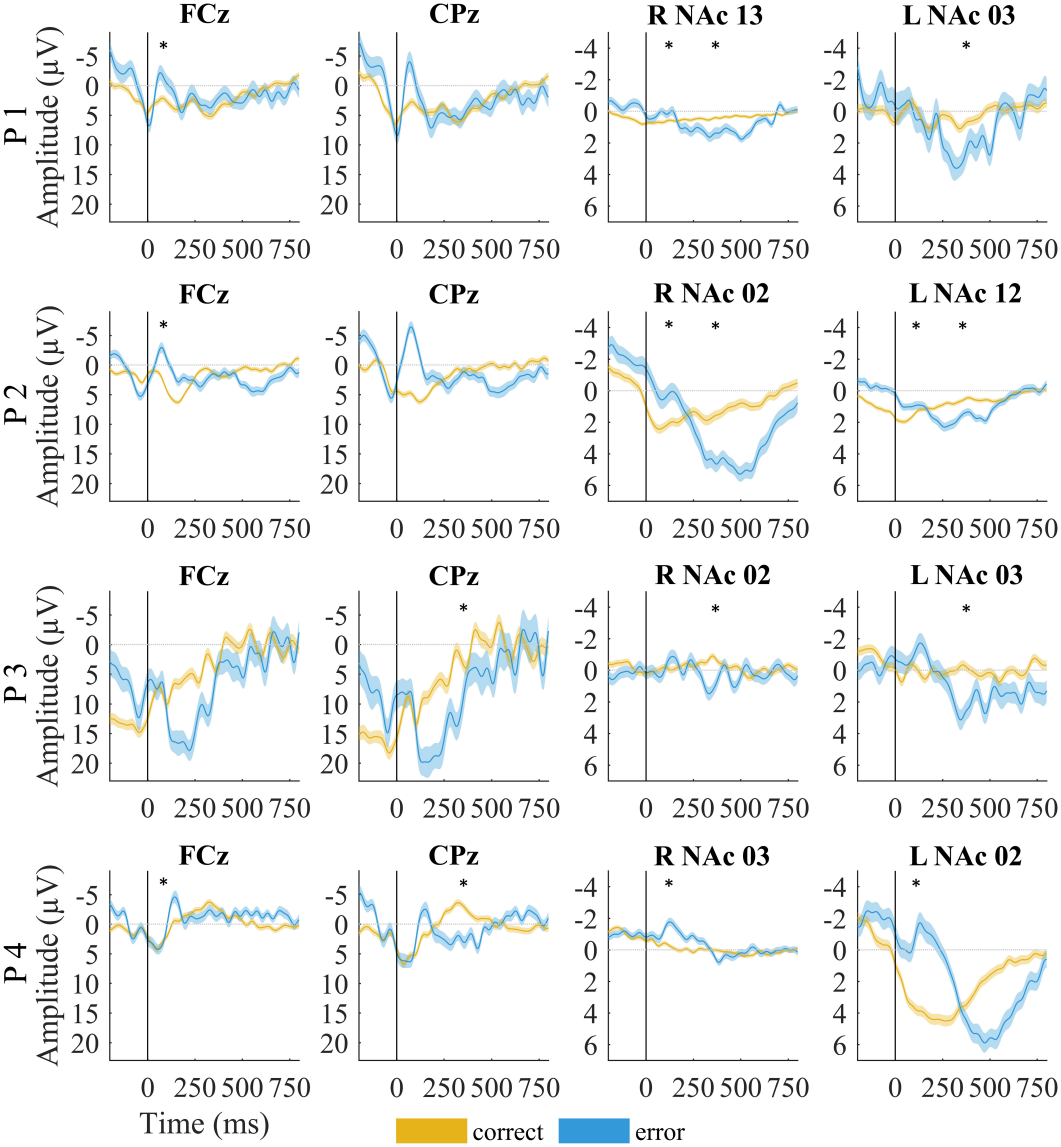
EEG and local field potentials of patients for error (yellow) vs. correct (blue) trials. The shaded lines indicate standard error of the mean. Asterisks mark significant amplitude differences between error and correct trials at the time of the error-related negativity/correct-related negativity and error positivity. Data were filtered with a 20-Hz low-pass filter for visualization.

The mean amplitude of the negative deflection was significantly larger for ERN than CRN for all but P3 (*p* = 0.73; all other *p* < 0.01, *t*-test; Table 2; Figure 3). The Pe amplitude was significantly larger in error than in correct trials for P3 and P4 (all *p* < 0.001, *t*-test; Table 2; Figure 3). This difference was not significant for P1 (*p* = 0.92) and P2 (*p* = 0.15).

**Table 2:** Amplitude measures of the error-related negativity (ERN)/correct-related negativity (CRN) at FCz and the error positivity at CPz.

	**ERN/CRN (μV)**				**Pe (μV)**			
	**Correct**	**Error**	***t (df)***	***p***	**Correct**	**Error**	***t* (*df*)**	***P***
P1	2.3 ± 0.5	−1.1 ± 1	−2.7 (367)	0.011	3.9 ± 0.4	4.0 ± 0.9	0.1 (367)	0.92
P2	1.4 ± 0.3	−1.3 ± 0.8	−3.4 (382)	0.002	1.1 ± 0.3	2.3 ± 0.6	1.6 (382)	0.15
P3	7.7 ± 0.6	7.2 ± 1.4	−0.4 (382)	0.73	2.2 ± 0.5	9.0 ± 1.3	5.3 (382)	<0.001
P4	0.1 ± 0.4	−3.0 ± 0.7	−3.8 (349)	<0.001	−1.6 ± 0.4	2.2 ± 0.6	5.2 (349)	<0.001

*Depicted are means (SEM) and t-test results*.

#### LFPs

Although the LFPs appeared to be heterogeneous at first sight, some activity patterns could be identified at the time of the ERN/CRN and Pe. ERN-LFPs were significantly more negative than CRN-LFPs bilaterally in P2 and P4 and showed a trend level difference in the right NAc in P1 (P1 right *p* = 0.063; P2 and P4 *p* >0.002; Table 3; Figure 3). Pe-LFPs were significantly more positive in error than in correct trials bilaterally in three patients (all but P4 *p* < 0.005).

**Table 3:** Amplitude measures of local field potentials (LFPs) in the right and the left NAc.

		**Error-related negativity-LFP (μV)**				**Pe-LFP (μV)**			
		**Correct**	**Error**	***t* (*df*)**	***p***	**Correct**	**Error**	***t* (*df*)**	***p***
Right NAc	P1	0.6 ± 0.1	0.2 ± 0.2	−2.0 (73)	0.06	0.4 ± 0.1	1.2 ± 0.3	3.3 (367)	0.002
	P2	2.2 ± 0.3	0.01 ± 0.5	−3.5 (382)	0.001	1.3 ± 0.2	3.6 ± 0.4	5.1 (130)	<0.001
	P3	−0.1 ± 0.2	0.2 ± 0.3	0.7 (382)	0.48	−0.4 ± 0.1	0.4 ± 0.3	3 (382)	0.0045
	P4	−0.2 ± 0.1	−1.5 ± 0.2	−5.4 (349)	<0.001	0.1 ± 0.1	−0.04 ±0.1	−0.6 (349)	0.58
Left NAc	P1	−0.4 ± 0.3	−0.3 ± 0.7	0.2 (367)	0.87	0.5 ± 0.2	2.1 ± 0.5	3.6 (367)	<0.001
	P2	1.8 ± 0.1	0.9 ± 0.2	−3.3 (382)	0.002	0.7 ± 0.1	1.7 ± 0.2	5.5 (382)	<0.001
	P3	0.01 ± 0.2	−0.5 ± 0.5	−1 (382)	0.44	0.1 ± 0.1	1.6 ± 0.4	3.8 (91)	<0.001
	P4	3.7 ± 0.3	−1.3 ± 0.6	−7.7 (349)	<0.001	3.2 ± 0.3	2.9 ± 0.4	−0.6 (164)	0.56

*Depicted are means (SEM) and t-test results*.

### Correlation Analysis

We found a significant correlation between the ERN and the ERN-LFP amplitudes unilaterally in two patients (all *p* < 0.002, *r* = 0.36 and *r* = 0.45; Table 4). The CRN and CRN-LFP correlated significantly bilaterally in P3 and unilaterally in three patients (all *p* < 0.002, 0.18 < *r* < 0.23). The Pe and Pe-LFP correlated significantly bilaterally in two patients and unilaterally in P1 (P4 showed a trend level difference at *p* = 0.052; all other *p* < 0.04, *r* = 0.23 and *r* = 0.55).

**Table 4:** Spearman correlation of the error-related negativity (ERN), correct-related negativity (CRN) and Pe with the respective local field potentials in the NAc.

		**ERN**		**CRN**		**Pe**	
		***r***	***p***	***r***	***p***	***r***	***p***
Right NAc	P1	−0.05	0.74	−0.003	0.95	0.42	0.005
	P2	0.23	0.19	0.23	<0.001	0.33	0.006
	P3	0.21	0.16	0.21	<0.001	0.49	<0.001
	P4	0.15	0.18	0.07	0.33	0.16	0.16
Left NAc	P1	0.01	0.93	0.18	0.002	−0.11	0.44
	P2	−0.12	0.41	−0.05	0.35	0.28	0.037
	P3	0.45	<0.001	0.19	0.001	0.55	<0.001
	P4	0.36	0.002	0.21	0.002	0.23	0.052

## Discussion

We explored performance monitoring processes in the NAc and MFC in patients with opioid abuse disorder performing a flanker task. Specifically, we intended to examine whether NAc modulation is related to well-known EEG potentials, the CRN, ERN, and Pe. We observed a significant differentiation between correct and error trials at the time of response in the NAc and a Pe-like deflection following errors in the NAc. Within-subject correlations between cortical and striatal potentials support the notion of an association of both structures in performance monitoring.

The NAc is widely acknowledged as a limbic–motor interface (Mogenson et al., 1980) and therefore a crucial structure for goal-directed behavior (Goto and Grace, 2005; Grace et al., 2007). It is structurally and functionally connected to central regions of the performance monitoring system, including the prefrontal cortex (for a review, see Ullsperger et al., 2014a; Salgado and Kaplitt, 2015). Accordingly, associations of NAc function with the ERN have been proposed (Ullsperger and von Cramon, 2006; Munte et al., 2007; Cohen et al., 2009a,b; Heinze et al., 2009; Kuhn et al., 2011). In line with those findings, we observed potentials related to error response in the NAc. In two of four patients, we found a bilateral correlation between these amplitude modulations and MFC recordings, indicating a functional relationship that, however, needs further confirmation.

Moreover, NAc activity has been linked to another important function of the performance monitoring system, the coding of uncertainty about action outcomes and subsequent behavioral adaptations (Berns et al., 2001; Buzzell et al., 2016; Hebart et al., 2016). Uncertainty about current task performance may be reflected by the CRN (Pailing and Segalowitz, 2004; Bartholow et al., 2005). We observed a significant NAc/MFC correlation for the CRN in five out of eight contrasts, indicating a functional interrelation between cortical and striatal processing that might be related to uncertainty about task performance.

Furthermore, a Pe-like deflection has previously been observed in the NAc (Munte et al., 2007) and in the subthalamic nucleus (Siegert et al., 2014), in line with our current findings. The results of the correlation analyses support the notion that this deflection is functionally related to the cortical Pe. Accordingly, our results indicate an important relationship between MFC and NAc that is likely linked to subjective error awareness and subsequent behavioral adjustments (Cohen et al., 2009c; Axmacher et al., 2010).

Nonetheless, some limitations of this study need to be considered. First, the study was performed with patients with long-lasting opioid abuse disorder. Opioid abuse disorder is associated with a hypo-activity within the cortico-striatal-thalamic loop, leading to impairments in cognitive control, such as decreased inhibition and conflict resolution (Yang et al., 2009). Accordingly, decreased amplitudes were reported in patients with heroin abuse disorder (Chen et al., 2013). Moreover, all patients were treated with levomethadone at the time of measurements, which can interfere with cognitive functions (for a review, see Wang et al., 2013). The sessions were also conducted 1–2 days following DBS surgery, and the resulting fatigue may have had an effect on behavioral performance. Considering these limitations, our results have to be treated with caution regarding the generalization to NAc/MFC function in a healthy population. Second, since LFPs can extend by several millimeters (Lempka and McIntyre, 2013), we cannot conclude whether the recorded LFPs exclusively represent activity from the NAc. However, the included channels are generally in line with a NAc source. Third, some correlations between cortical and striatal activity reached significance, although one (or both) of the potentials did not discriminate errors and correct responses. These correlations have to be considered as unrelated to response accuracy processing.

Overall, our study demonstrates that NAc activity is modulated by response accuracy at the time of cortical ERN/CRN and Pe, providing support for its role in performance monitoring. Moreover, cortical and NAc potential amplitudes were interrelated, supporting the notion that MFC and NAc are functionally interrelated parts of the performance monitoring network. Our results align with previous studies using imaging (Rodriguez et al., 2006; Simões-Franklin et al., 2010) and electrophysiological (Munte et al., 2007; Kuhn et al., 2011; Cohen et al., 2012) techniques that report on the involvement of the NAc in performance monitoring network. Consequently, modulating this network through DBS might alter impairments caused by a psychiatric disorder (Kuhn et al., 2011; Figee et al., 2013). Specifically, striatal stimulation might be able to improve impaired performance monitoring, and this effect might possibly be reflected by changes in EEG correlates (Kuhn et al., 2011; Figee et al., 2013; Smolders et al., 2013). This should be directly investigated by future studies comparing EEG markers pre-surgery and on/off stimulation and exploring their potential interrelation with clinical improvement during DBS treatment. Concerning the observed cortico-striatal interrelation in this study, this electrophysiological marker might provide first clues toward a performance monitoring correlate that can be used to guide close-loop stimulation. Considering the limitations of this study, further research is needed to confirm our results and advance our understanding of NAc/MFC functions in performance monitoring.

## Data Availability Statement

The raw data supporting the conclusions of this article will be made available by the authors, without undue reservation.

## Ethics Statement

The studies involving human participants were reviewed and approved by Ethics Committee of the University of Cologne, Faculty of Medicine and University Hospital Cologne, Cologne, Germany. The patients/participants provided their written informed consent to participate in this study.

## Author Contributions

JK and MU designed the research. ES and TS performed the research. TG, VV-V, and DH contributed unpublished reagents/analytic tools. ES analyzed the data and wrote the manuscript. All the authors edited the manuscript.

## Conflict of Interest

The authors declare that the research was conducted in the absence of any commercial or financial relationships that could be construed as a potential conflict of interest. The reviewer JK declared a shared affiliation, though no other collaboration, with several of the authors ES, TS, VV-V, DH, and JK to the handling Editor.
